# Modeling and analysis of the impacts of jet lag on circadian rhythm and its role in tumor growth

**DOI:** 10.7717/peerj.4877

**Published:** 2018-06-06

**Authors:** Azka Hassan, Jamil Ahmad, Hufsah Ashraf, Amjad Ali

**Affiliations:** 1 Research Center for Modeling and Simulation (RCMS), National University of Scinces and Technology (NUST), Islamabad, Pakistan; 2 Atta-ur-Rahman School of Applied Biosciences (ASAB), National University of Science and Technology, Islamabad, Pakistan

**Keywords:** Circadian clock, Jet lag, Tumor progression, Model checking, Petri net, Qualitative modeling

## Abstract

Circadian rhythms maintain a 24 h oscillation pattern in metabolic, physiological and behavioral processes in all living organisms. Circadian rhythms are organized as biochemical networks located in hypothalamus and peripheral tissues. Rhythmicity in the expression of circadian clock genes plays a vital role in regulating the process of cell division and DNA damage control. The oncogenic protein, MYC and the tumor suppressor, p53 are directly influenced by the circadian clock. Jet lag and altered sleep/wake schedules prominently affect the expression of molecular clock genes. This study is focused on developing a Petri net model to analyze the impacts of long term jet lag on the circadian clock and its probable role in tumor progression. The results depict that jet lag disrupts the normal rhythmic behavior and expression of the circadian clock proteins. This disruption leads to persistent expression of MYC and suppressed expression of p53. Thus, it is inferred that jet lag altered circadian clock negatively affects the expressions of cell cycle regulatory genes and contribute in uncontrolled proliferation of tumor cells.

## Introduction

The environment around us is under constant change. Some of these changes are unique while others repeat in a timely manner for example day and night and seasonal changes. To accommodate these diverse changes, an organism adapts itself to new environment in a rhythmic manner. This results into evolving the time keeping mechanism of organisms that allows them to habituate the external environmental changes. With every new day, human body resets all the biological and physiological activities for the daily routine and each process starts to oscillate in a timely manner (Panda, Hogenesch & Kay, 2002). This endogenous oscillation has been termed as “circadian rhythm” which has been derived from the Latin phrase “circa diem” (meaning “about a day”) (Greene, 2012; Savvidis & Koutsilieris, 2012; Leloup & Goldbeter, 2004). Circadian oscillations are self-supported and endogenous time keeping systems. They oscillate in accordance with a 24 h routine that enables organisms to survive environmental changes, thereby familiarizing their activities to the appropriate time of day (Savvidis & Koutsilieris, 2012; Leloup & Goldbeter, 2004; Masri, Cervantes & Sassone-Corsi, 2013; Sahar & Sassone-Corsi, 2009). Circadian oscillations require entrainment by the external environment without which they dissociate from the natural cycles (Greene, 2012). One of the most powerful stimulus is the light/dark cycle which not only regulates the sleep/wake cycle but also controls other hormonal secretions and metabolic processes (Greene, 2012; Sahar & Sassone-Corsi, 2009; Golombek & Rosenstein, 2010).

### The circadian clock

Several studies (Greene, 2012; Dibner, Schibler & Albrecht, 2010; Yamamoto et al., 2004; Kalsbeek et al., 2011; Mohawk, Green & Takahashi, 2012; Damiola et al., 2000) have categorized circadian clock into central and peripheral domains. The master clock, which is also known as suprachiasmatic nucleus (SCN), is located in the anterior hypothalamus. It is a paired structure where each part contains approximately 10,000 neurons. SCN receives visual signals as external stimuli and other non-photic signals through different hormonal and neuronal tracts. SCN as a master clock or synchronizer has a duty to transmit timekeeping signals to other parts of the body (Greene, 2012; Dibner, Schibler & Albrecht, 2010; Kalsbeek et al., 2011). Peripheral clocks are present in the different organs such as liver, kidney, pancreas, thyroid gland (Yamamoto et al., 2004), etc. These peripheral clocks are entrained directly from the SCN through different signaling mechanisms which involve circulating hormones, metabolites and neuronal signals (Yamamoto et al., 2004; Yamazaki et al., 2000; Oster et al., 2006). However, there are a number of other external factors like daily feeding/fasting routine and temperature which are responsible for the entrainment (Damiola et al., 2000). As this autonomous clock has been found to be omnipresent, almost every cell in the body maintains a rhythmicity in its functions (Zhang et al., 2014). Experimental studies (Mohawk, Green & Takahashi, 2012) have shown that each cell responds variably to entraining signals and controls different physiological outputs. The mitotic and gating activities during cell division also follow a rhythmic oscillatory pattern (Matsuo et al., 2003).

### Molecular mechanism of circadian clock

**Figure 1:**
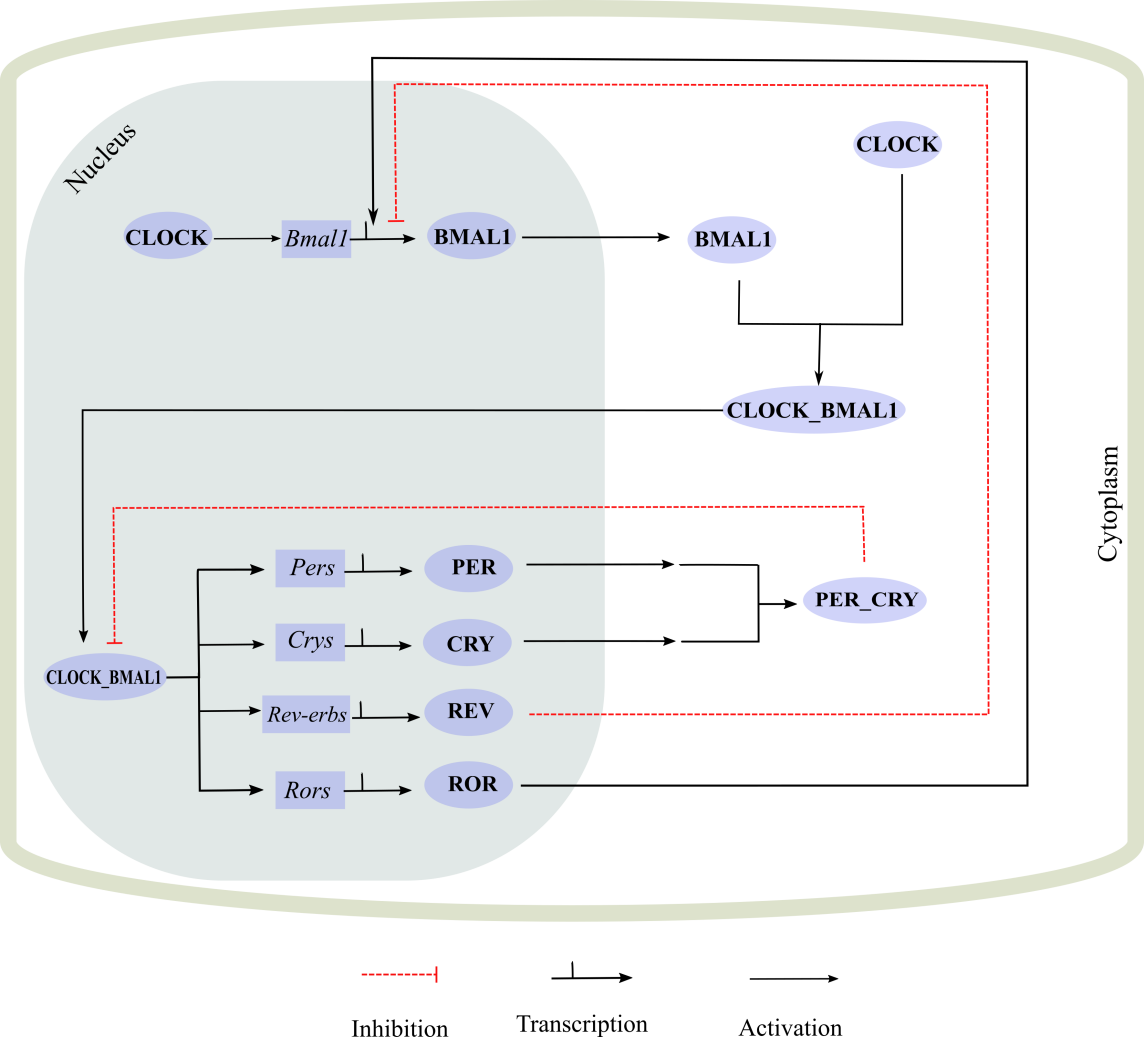
A simplified representation of molecular machinery of the circadian clock. The circadian clock consists of positive and negative interconnecting transcription/translational feedback loops which control circadian timings. *Bmal1* transcription is initiated by CLOCK protein and both heterodimerize to form a complex. The CLOCK-BMAL1 complex (positive regulators) serves as the activator and prompts the transcription of *Per* and *Cry* genes. PER and CRY proteins then heterodimerize forming a PER-CRY complex. This complex inhibits the CLOCK-BMAL1 function, thus leading to the suppression of its own transcription. CLOCK-BMAL1 also controls the regulation of the nuclear receptors *Rors* and *Rev-erbs*, which help in regulating *Bmal1* transcription.

At the molecular level, circadian clock mechanism in both core and the peripheral clocks is known to be analogous. This mechanism comprises of a complex system of translational and transcriptional feedback loops that oscillate in a 24 h manner (Reppert & Weaver, 2002; Lee et al., 2001; Shearman et al., 2000). The mechanism revolves around two coupled protein complexes. The first one comprises of CLOCK (Circadian Locomotor Output Cycles protein Kaput) along with BMAL1 (Brain and Muscle ARNT like receptor 1) and the second consists of PER (Period) proteins with CRY (Cryptochrome) proteins. CLOCK-BMAL1 complex plays its part as a positive limb, i.e., as an activator and the second complex PER-CRY acts as the negative limb of the cycle i.e., as an inhibitor of the CLOCK-BMAL1 complex. This cycle works in such a manner that CLOCK activates the transcription of BMAL1 and then they heterodimerize which leads to the formation of CLOCK-BMAL1 complex. This complex then activates the transcription of several genes, of which the most important are *Pers* and *Crys*. PER and CRY proteins then heterodimerize and exert a negative impact on the CLOCK-BMAL1 complex, thus suppressing its function and their own transcription indirectly. An additional linked negative feedback loop is working at the same time in which CLOCK-BMAL1 activates nuclear receptors, RORs and REV-ERBS and after activation, RORs regulates *Bmal1* transcription whereas REV-ERBS inhibits it. A pictorial example of the whole mechanism is shown in Fig. 1. Every cycle of these inter connected processes takes about 24 h, thus enabling the clock to regulate other processes at proteomic or metabolic level at specific times of the day (Greene, 2012; Shearman et al., 2000; Ko & Takahashi, 2006; Reddy et al., 2005; Yang et al., 2009).

### Link between circadian clock and cellular proliferation

Another essential oscillatory system in our body is the cell cycle which allows a cell to reproduce efficiently and safely. This cycle starts with a mother cell going through different phases of enlargement and replication of DNA. The earlier stages in which the cell is being prepared for mitosis are known as interphase. After the cell has been prepared, it enters the mitotic phase for division in two daughter cells and after that the cycle continues. Cell cycle is highly regulated to ensure cell viability to detect any DNA damage within the cell for preventing it to daughter cells. Molecular checkpoints control the progression of a cell and ensures the activation of DNA repair pathways in order to eliminate any DNA damages (Schibler, 2003; Bolsover et al., 2011).

**Figure 2:**
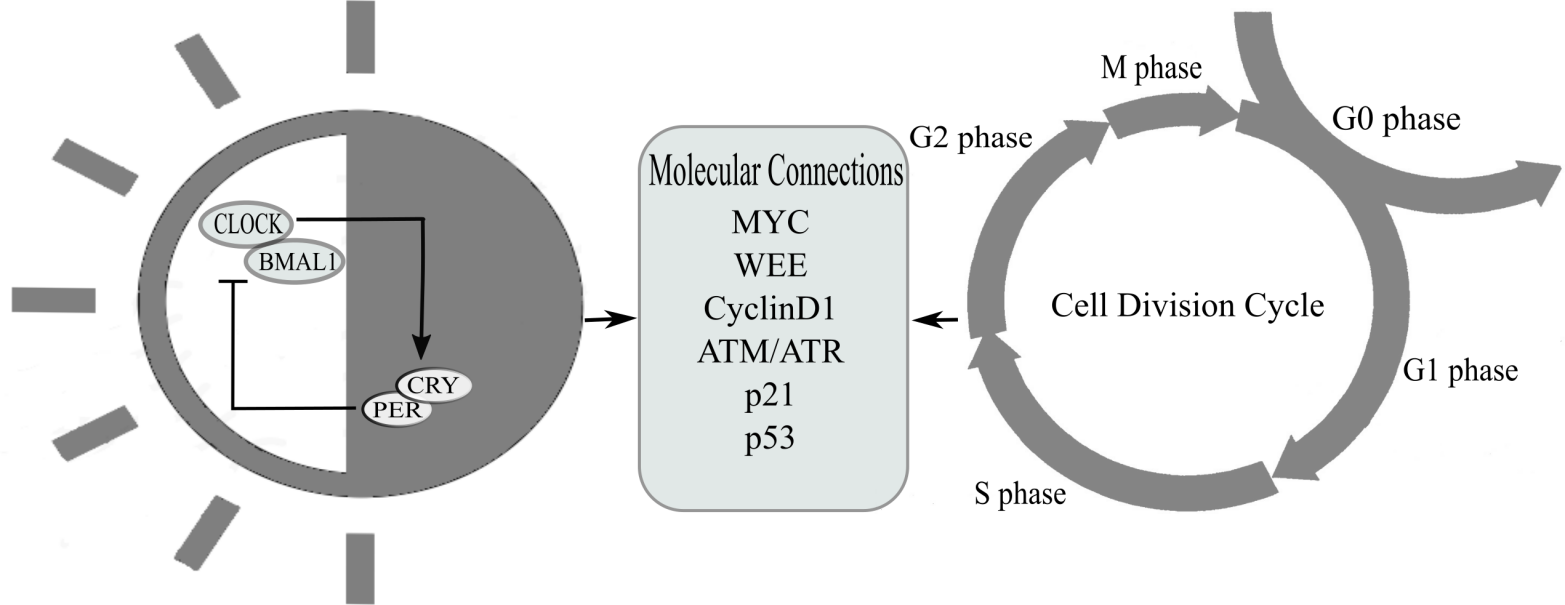
This figure represents proteins that are coupled between cell cycle and circadian clock. The circadian system is linked to the cell-division cycle through regulation of gene expression and post-translational mechanisms. Proteins regulating cellular proliferation, such as Wee1, Myc, Cyclin D1 and those involved in different cell division checkpoints for initiating DNA repair pathways such as p21, p53 and ATM/ATR, are under circadian influence.

Many experimental studies have reported that circadian genes are involved in the regulation of cell division (Greene, 2012; Savvidis & Koutsilieris, 2012; Masri, Cervantes & Sassone-Corsi, 2013; Reddy et al., 2005; Rana & Mahmood, 2010; Li, Ao & Wu, 2013). It has been observed that CLOCK-BMAL1 complex can directly regulate cell cycle genes through E-box mediated reactions which is the binding site for CLOCK-BMAL1 complex. A number of cell-cycle genes contain E-boxes in their promoter regions (Rana & Mahmood, 2010; Matsuo et al., 2003). Cell cycle genes under direct influence of circadian clock include *Wee1, Myc, Cyclin D1, p53* (see Fig. 2). They play an important role at different checkpoints of cell division and are in involved in cell proliferation and damage control (Matsuo et al., 2003; Fu et al., 2002; Oishi et al., 2003). These cell cycle regulatory genes appear to be under direct circadian influence and are disturbed due to any disturbance in the circadian clock (Sahar & Sassone-Corsi, 2009; Matsuo et al., 2003; Canaple, Kakizawa & Laudet, 2003; Filipski et al., 2004).

Genes and proteins that appear to be ablated in cancer cells are under direct influence of the circadian clock. *Myc* plays an important role in apoptosis and cell proliferation. It is an oncogene, therefore, its deregulation can result in uncontrolled cell proliferation and genomic instability (Vita & Henriksson, 2006). Alteration of normal cells into tumors is facilitated by MYC protein which must be overcome by p53 through its apoptotic action (You et al., 2002; Evan & Vousden, 2001). p53 is a tumor suppressor protein and plays an important role at G1-S checkpoint (Shaw, 1996). Oncogenic transformation initiated by MYC must be controlled (You et al., 2002) in the cell in which p53-mediated apoptosis plays an important part (Evan & Vousden, 2001; Vafa et al., 2002).

### Circadian disruption due to jet lag and its role in cancer

One of the major life style changes involve abnormalities in sleep/wake cycle due to night shifts and frequent traveling, which generate the effect of jet lag (Savvidis & Koutsilieris, 2012). Jet lag is known as circadian desynchrony, a sleep disorder caused by traveling across several time zones resulting in misalignment between internal circadian clock and the destination’s local time. Inability of an individual to adapt to a sudden shift in these synchronizers causes a desynchronization between the body and the external environment. Its severity depends on several variables including the direction of travel and the time zones crossed. Studies (Lee et al., 2010; Zee, Attarian & Videnovic, 2013; Choy & Salbu, 2011) show that long term frequent time zone changes cause physiological and psychological health issues. Shift work also produces a similar effect as jet lag due to irregular sleep/wake routine and exposure to light at unusual times. Disruption of circadian rhythms due to these is associated with various forms of cancer in humans. Epidemiological studies (Davis & Mirick, 2006; Conlon, Lightfoot & Kreiger, 2007; Band et al., 1996; Hansen, 2006; Keith, Oleszczuk & Laguens, 2001; Pukkala et al., 2003) report that there is an increased incidence of cancers in pilots and flight attendants due to frequent trans-meridian flights. These studies suggest that the consistent and long term disturbances in light/dark and sleep/wake cycle lead to the disruption of circadian clock which can make body tumor prone. Similarly, people working in night shifts have a considerably higher risk of developing breast cancer, colon cancer, endometrial cancer, prostate cancer and non-Hodgkin lymphoma. Several studies (Savvidis & Koutsilieris, 2012; Filipski et al., 2004; Sahar & Sassone-Corsi, 2009; Zhu et al., 2008; Hoffman et al., 2009; Chu et al., 2008) prove that chronic circadian rhythm disruption plays an important role in making the body cancer prone. A number of experimental studies (Sahar & Sassone-Corsi, 2009; Filipski & Lévi, 2009; Fu et al., 2002; Lee et al., 2010) on animal models prove jet lag disruption of circadian rhythms. This disruption facilitates tumor growth as circadian clock directly regulates cellular proliferation and repair. Due to disrupted circadian rhythms, cell division cycle becomes less efficient in DNA repair and proliferate abnormally which result in tumor development.

### Computational analysis of clock disruption and cancer

A system-level understanding of a biological network can give us various important insights into the dynamics of the system. Studying a network of gene interactions and biochemical pathways can help in understanding a system’s behavior over time under various conditions. It can also help to determine the control mechanisms which can be used to minimize the malfunctions and can also provide potential therapeutic targets for the treatment of disease (De Jong, 2002). The understanding of the link between circadian clock disruption, cell cycle disturbance and cancer, based on wet lab experimental data has been established in several studies (Sahar & Sassone-Corsi, 2009; Lee et al., 2010). In this study, computational techniques have been employed to study the mechanism of jet lag mediated disruption in circadian rhythms that lead to tumor growth. The system has been modeled in its abstracted form while preserving the experimentally verified behavior of the entities. Analysis of the simulation has been performed to study the behavior of the circadian system and its role of disruptions in tumor progression.

### Our contribution

To understand the mechanism and the oscillatory behavior of circadian clock, several models (Becker-Weimann et al., 2004; Leloup & Goldbeter, 2004; Paetkau, Edwards & Illner, 2006; Strogatz, 1987; Sriram, Bernot & Képès, 2006) utilizing mathematical modeling technique and graph based modeling approach have been constructed. These models help in understanding the feedback mechanism and oscillatory behavior of the circadian system. Other mathematical models focus on studying the desynchronization of circadian clock due to jet lag and observe the space dynamics to look at eastward to westward severity of jet lag effects on circadian clock (Kori, 2015; Kori, Yamaguchi & Okamura, 2017; Lu et al., 2016). In this study, we have used graph based modeling to model the interaction between circadian clock and vital proteins MYC and p53 which are involved in cellular proliferation. This model depicts connection of circadian clock to cell cycle at a molecular level. We analyze the behavioral changes in the oscillations of circadian clock due to jet lag and also observe the negative impacts of these changes on cellular proteins. As this model presents a link between jet lag, circadian misalignment and tumor progression, therefore, the model provides valuable insights into the dynamics of system which can be useful in chronotherapies in future.

## Methods

**Figure 3:**
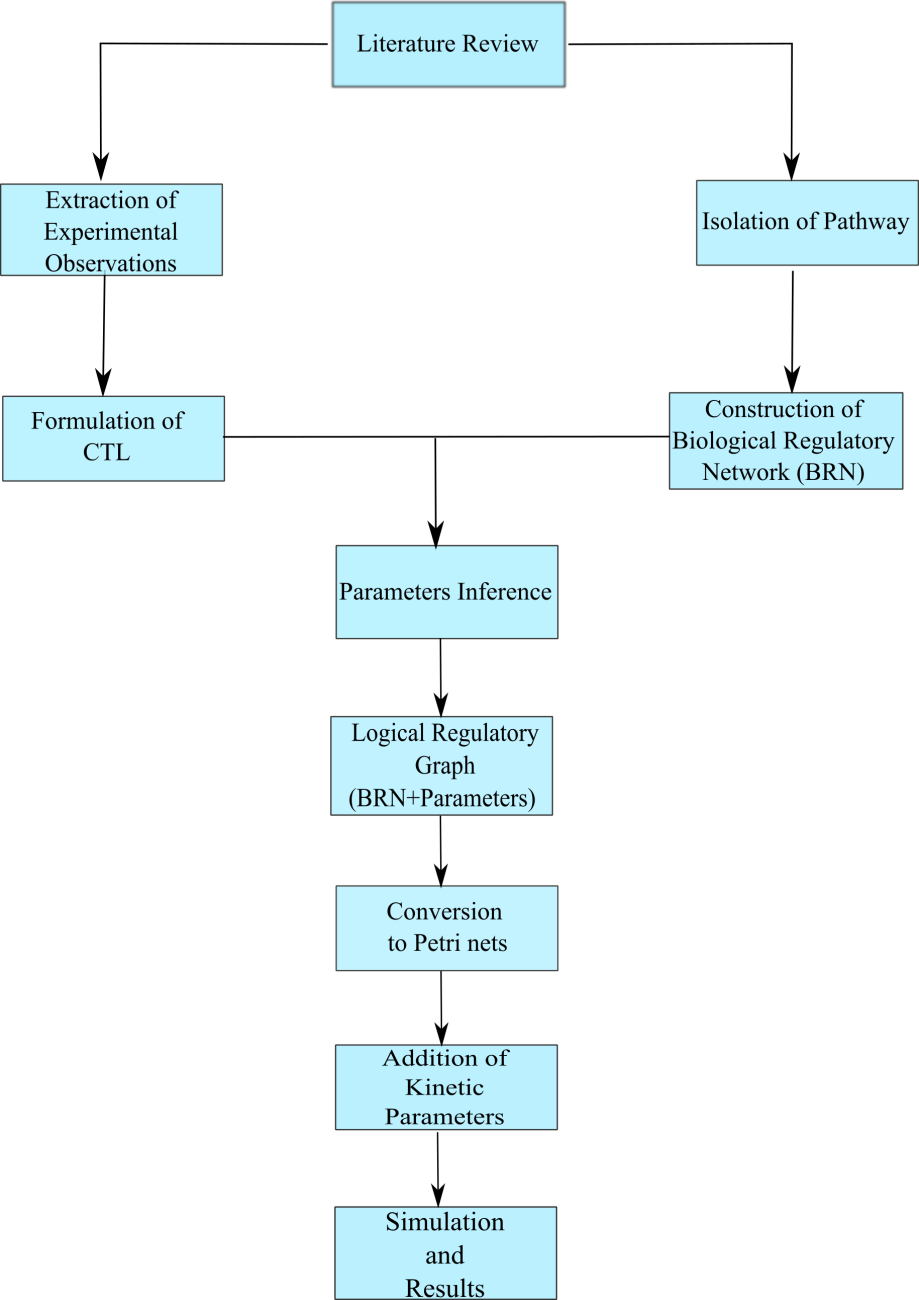
The workflow employed in this study.

In this section, the methodology employed in this study has been discussed. A schematic work flow of this methodology framework has been shown in Fig. 3.

The work flow starts with literature review followed by abstraction of pathway and extraction of the important experimental observations that were then encoded into Computation Tree Logic (CTL) formulas (see Supplementary File 3). The Biological Regulatory Network (BRN) and CTL formulas were used to infer the logical parameters using SMBioNet (Khalis et al., 2009; Richard, Comet & Bernot, 2006). A Logical Regulatory Graph which is the combination of BRN and logical parameters was implemented in the software GINsim (Naldi et al., 2009) (see Supplementary File 2). This logical regulatory graph was then converted into Petri net framework using the export option available in GINsim. The exported standard Petri net was converted into Timed Continuous Petri net using the software Snoopy (Heiner et al., 2012). This Petri net was modified by assigning rates and delays to transitions based on biological observations (see Supplementary File 1).

### René Thomas’ logical formalism

**Figure 4:**
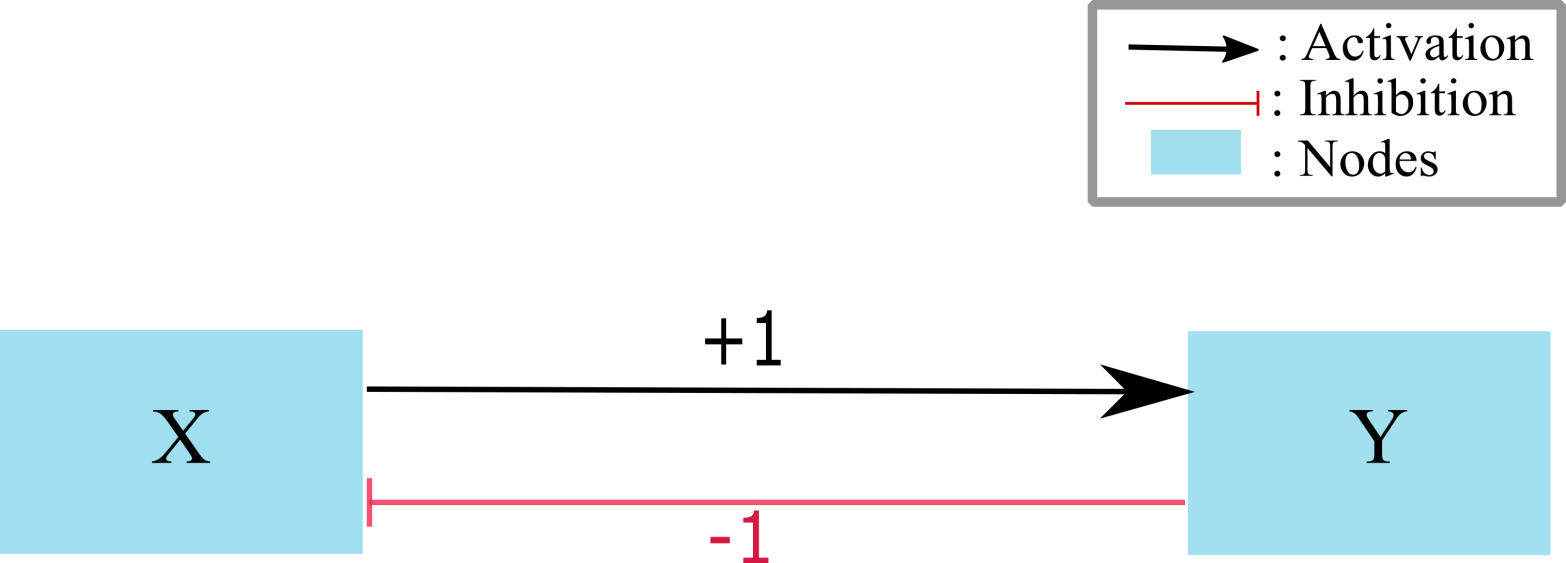
A toy BRN with two entities *X* and *Y*, where *X* is activating *Y* (shown by the edge labeled with +1) and *Y* inhibiting *X* (shown by an edge labeled with −1).

In the late 1970s, René Thomas presented kinetic logic formalism for qualitative modeling of Biological Regulatory Networks (BRNs) (Thomas, 1991). This graph based formalism has its benefits over other boolean formalisms due its ability to allow interaction threshold levels above “1”. It has been proved that Kinetic Logic can capture the the dynamics in similar way to differential equations, however, it keeps the system less complex due to discretization (Thomas, 1991) of expression levels. Moreover, it allows asynchronous dynamics to model cyclic trajectories which was not possible in the synchronous boolean formalism (Kauffman, 1969; Inoue, 2011). Thomas’ formalism uses graph theory to model Biological Regulatory Networks (BRNs). The components of a BRN include entities and the interactions among them. The expressions of an entity are shown by discrete levels and their interactions are threshold dependent, i.e., once the threshold is reached the interaction can takes place (see Fig. 4). The semantics of Kinetic Logic Formalism is based on Graph Theory. We adopt the semantics of this formalism from different studies (Ahmad et al., 2012; Bernot et al., 2004; Thomas, 2013; Ahmad et al., 2006).

Definition 1 **Directed Graph**

*A graph G* = (*V*, *E*)* is a tuple where:*

• V represents the set of vertices
• *E*⊆*V* × *V represents the set of edges (ordered pairs of vertices).*

Definition 2 **Biological Regulatory Network**

*A biological regulatory network is a labeled directed graph G*(*V*, *E*)* where V is the set of biological entities and E*⊆*V* × *V is the ordered set of directed interactions among them. Each edge* (*v*_*i*_*, v*_*j*_*) has a pair* (*l*, *t*_*v*_*i*_,*v*_*j*__)* as its label where l is the sign of interaction (‘+’ for activation and ‘−’ for inhibition) and t*_*v*_*i*_,*v*_*j*__ ∈ {1, 2, ..., *r*_*vi*_}* is the threshold of the interaction where r*_*v*_*i*__* is less than or equal to the out-degree of v*_*i*_*.*

All edges of a BRN are labeled according to the threshold level and type of interaction (as an example see Fig. 4). The resources of an entity depends on the presence and absence of its activators or inhibitors at any instant of time. In Fig. 4, when *X* = 1 then it is the resource of *Y* and when *Y* = 0 then it is the resource of *X* (the absence of inhibitor is treated as a resource). The discrete expression levels of an entity is the set containing the integers 0 to its highest threshold in the BRN. For example, the expression levels of *X* and *Y* is the same set {0, 1} as both have their highest thresholds equal to 1. A state of a BRN is an element of the Cartesian product of the sets of expression levels of all entities. The states of the toy BRN are given in the set {(0, 0), (0, 1), (1, 0), (1, 1)}. Each state determines the level of an entity evolving in the state space. A state space defines all possible configurations of entities represented by a state graph (qualitative model). State graph is generated against a particular set of logical parameters determining the behavior of entities in that particular state. A Logical parameter is represented by *K*_*entity*_{*resources*} and it is the functions of resources of an entity. The values of a *K*_*entity*_{*resources*} parameter always lie in the set {0, ..., *j*} where *j* is less than or equal to the highest threshold of the entity. The values of these parameters are unknown a priori (Ahmad et al., 2012; Bernot et al., 2004; Thomas, 2013; Ahmad et al., 2006). For the parameters *K*_*X*_{} = 0, *K*_*X*_{*Y*} = 1, *K*_*Y*_{} = 0 and *K*_*Y*_{*X*} = 1, the state graph of the toy BRN is a closed path (cycle): (0, 0) → (1, 0) → (1, 1) → (0, 1) → (0, 0).

#### Construction of logical regulatory graph

For construction of a logical regulatory graph based on René Thomas’ logical formalism, the so-called software tool GINsim (Naldi et al., 2009) was used. Two main types of graphs are constructed and generated with the help of GINsim: Logical Regulatory Graph which comprises of a BRN and its logical parameters and State Transition Graphs (State Graph) which represents the dynamical behavior of entities.

### Model checking approach to infer K-parameters

The logical parameters of a BRN should be consistent with wet-lab experiments/ observations. They help us to understand the dynamics of a BRN. The formal methods based automatic model-checking technique can be employed for the computation of parameters (Bernot et al., 2004). To check whether a property is verified or not in a state space, the model-checking technique exhaustively check the state apace of a model for the given property (Baier, Katoen & Larsen, 2008). Model-checking techniques verify properties which are formally expressed in temporal logic. Temporal Logic can either be Linear-time Temporal Logic (LTL) or Computation Tree Logic (CTL). As CTL can cater the branching time systems, therefore, it is preferred for biological networks. Wet-lab observations are first encoded in CTL and then verified in the state space of a BRN. State spaces are generated for all the possible combinations of logical parameters. Only those parameter sets are selected which satisfy the CTL formulas (Clarke, Grumberg & Peled, 1999). CTL formulas involve path and state quantifiers to represent the properties of the system. These formulas also supports complex forms like nesting of path-state quantifiers for verification of complex behaviors. These quantifiers are described as follows:

• Path Quantifiers: The two path quantifiers are ∃ and ∀, where ∀ specifies all paths originating from a current state and ∃ specifies at least one path originating from the current state.
• State Quantifiers: The state quantifier ‘□’ (globally) specifies that all the states along the specified path verify the property. The quantifier ‘♢’ (future) specifies that at least one future state along the specified path should hold the given property. The quantifier ‘○’ (next) specifies the first successor state(s) of the current state satisfy the property and ‘$\mathcal{U}$’ (until) specifies that a property holds (for example, *ϕ* in $\mathrm{&phi;} \mathcal{U} \mathrm{&psi;}$) until another property holds (for example, *ψ* in $\mathrm{&phi;} \mathcal{U} \mathrm{&psi;}$).

#### Software used for model checking

For the inference of parameters, the tool SMBioNet (Selection of Models of Biological Networks) (Khalis et al., 2009; Richard, Comet & Bernot, 2006) was utilized. It employs model checking approach to generate parameter sets satisfying the desired properties encoded in the form of CTL logic. The input file of SMBioNet consists of entities as variables, their interactions, ranges of *K* parameters and CTL formulas. For each possible set of parameters (from the their ranges), a state graph (qualitative model) exists. However, SMBioNet selects only those models which satisfy the properties (biological observations) encoded in CTL.

### Conversion of BRN to Petri nets

Petri nets were developed by Carl Adam Petri for the analysis of the concurrent processes occurring in technical systems (David & Alla, 2010; Brauer & Reisig, 2009; Blätke, Heiner & Marwan, 2011). However, due to its simplicity and flexibility it has been successfully applied in other domains as well, such as chemical reactions, biochemical processes etc.. This framework allows us to model discrete, continuous and hybrid systems. Petri nets have already been used for modeling several complex regulatory networks and pathways because of their versatility and ability to cater hybrid systems. Transcriptional, metabolic and protein-interactions can be modeled together as a single system (Scott et al., 2014; Liu & Heiner, 2013; Li & Yokota, 2009; Chaouiya, Remy & Thieffry, 2008; Formanowicz et al., 2007; Simao et al., 2005; Heiner, Koch & Will, 2004; Chaouiya, 2007).

GINsim allows to export the logical regulatory graph (BRN and K-parameters) into Petri net using the method described by Chaouiya, Naldi & Thieffry (2012). The following definition of Timed Continuous Petri net has been adapted from Tareen & Ahmad (2015).

Definition 3 **Timed Continuous Petri net (TCPN)**

*A Timed Continuous Petri net is a tuple* 〈*P*, *T*, *f*, *h*, *m*_0_, *tempo*〉 * where:*

• P is the finite set of places
• T is the finite set of transitions
• f: (*P* × *T*)∪(*T* × *P*) → ℝ_≥0_ *is the application that assigns positive real numbers (weights) to directed arcs*
• *h*: *T* → {*T*^*D*^, *T*^*C*^} *is the hybrid function that assigns the type ‘delayed’* (*T*^*D*^) *or ‘continuous’* (*T*^*C*^) *to each transition*,
• *m*_0_: P →ℝ_≥0_ *is the initial marking of positive real values of places,*
• *tempo*: *T* → {ℚ_≥0_|*t* ∈ *T*^*D*^}∪{ℚ|*t* ∈ *T*^*C*^} *is an assignment function that assigns delays to delayed (deterministic) transitions and rates to continuous transitions*.

**Figure 5:**
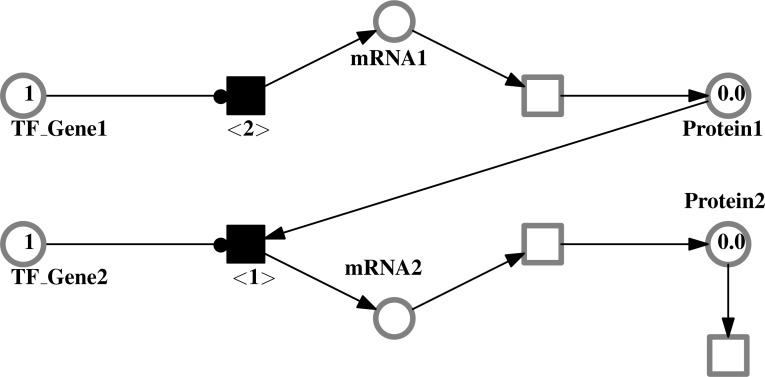
An example of Timed Continuous Petri net where ‘○’ represents places and ‘□’ represents transitions. All the places are continuous. Places named ‘TF_Gene1’ and ‘TF_Gene2’ represent Transcription factor of Gene1 and Gene2, respectively. Black filled transition represent ‘Transcription’, as ‘Delayed transition’, which control the time delays. The unfilled transitions represent ‘Translation’ as continuous transitions.

Example of a Timed Continuous Petri net is shown in Fig. 5.

#### Software used for Petri net construction

Snoopy (Heiner et al., 2012) was used in this study for Petri net construction and simulations. This tool allows many variants (discrete, timed, stochastic, continuous, hybrid and colored) of Petri nets as modeling frameworks to analyze systems effectively. Models can be hierarchically structured for the modeling of large systems. This tool has been successfully used for the modeling and analysis of many type of complex systems.

## Results

This section discuses the results including construction of BRN, inference of parameters, construction of a logical regulatory graph followed by its conversion to Petri net. As this study focuses on the tumor growth due to disturbed circadian clock, only proteins that are involved in tumor proliferation are studied.

### Construction of BRN

**Figure 6:**
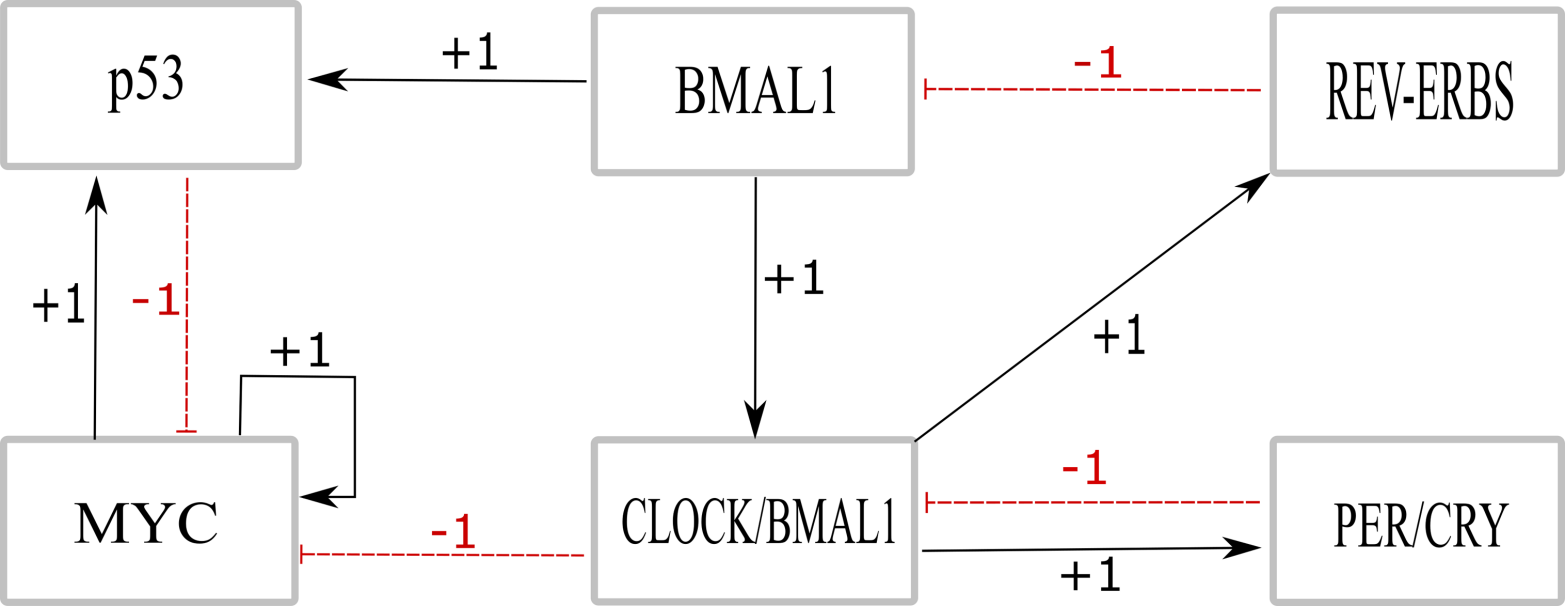
The BRN consisting of six entities involving the core clock proteins and proteins that are involved in tumor growth, i.e., p53 and MYC. There are four inhibitory interactions labeled with −1 and six activation interactions labeled with +1.

From Fig. 2 only those entities that are of concern in this study are included in the BRN shown in Fig. 6. The activation and inhibition interactions are also based on experimental observations. For example, BMAL1 forms a complex with CLOCK protein resulting in CLOCK-BMAL1 complex, i.e., it activates the formation of this complex. This has been shown in Fig. 6 by an activation edge from BMAL1 to CLOCK-BMAL1. Activation edges have been used from CLOCK-BMAL1 complex to PER-CRY complex and REV-ERBS, since the former complex is involved in the transcription of PER, CRY and REV-ERBS proteins. PER-CRY complex inhibits the transcription of CLOCK-BMAL1 complex and REV-ERBS inhibits the transcription of BMAL1 protein, therefore, in these cases an inhibitory edge is used (see Fig. 6) (Greene, 2012; Shearman et al., 2000; Ko & Takahashi, 2006; Reddy et al., 2005; Yang et al., 2009). MYC is inhibited by the CLOCK-BMAL1 complex and p53 to suppress its oncogenic activation (Lévi et al., 2007; Matsuo et al., 2003; Fu et al., 2002), which is represented by an inhibitory edge. BMAL1 is known to activate p53 tumor suppressor pathway (Jiang et al., 2016), therefore, an activation edge has been used from BMAL1 to p53. Increased levels of MYC generate oncogenic stress that activates tumor suppressor protein p53 (Hoffman & Liebermann, 2008). This is represented by an activation edge from MYC to p53. Myc protein is a transcription factor and activates the expression of many genes. Its own expression is regulated mainly by the mitogenic signals or growth signals. As soon as a cell receives mitogenic stimulus, Myc starts working. Thus, in normal cells its levels keep on fluctuating (with one maximum and one minimum level). However, expression of Myc becomes persistent in the absence of p53 which is a phenomenon that is observed in cancer cells. So the positive loop on Myc in the BRN ensures that it is activated repeatedly (as happens in a normal proliferating cell and its expression becomes persistent in the absence of tumor suppressor p53). Threshold levels of all the interactions were kept 1 to keep the system boolean.

### Inference of parameters

For the construction of a logical regulatory graph for the BRN shown in Fig. 6, logical parameters were estimated with the help of SMBioNet. Computation Tree Logic (CTL) was used to specify biological properties of the network. Formulas *ψ*_1_ to *ψ*_5_ were used in conjunction and the parameter sets satisfying these properties were selected.

(1) \begin{eqnarray*}{\psi }_{1}=\mathit{Init}\Rightarrow \forall \circ (\exists \diamond (\mathit{Init}))\end{eqnarray*}

Init (Initial state) in *ψ*_1_ represents (CB = 0 ∧ PC = 0 ∧ *R* = 0 ∧ Myc = 0 ∧ p53 = 0). This property states that the system is in a state where all entities are at zero level and later on the system arrives back to the same state. This property represents the homeostasis.

(2) \begin{eqnarray*}\begin{array}{@{}ll@{}} \displaystyle &\displaystyle {\psi }_{2}=\exists \diamond (\mathit{Bmal = 0}\wedge \mathit{Clock}\text{-}\mathit{Bmal1}=0\wedge \mathit{Per}\text{-}\mathit{Cry}=0\wedge \mathit{Rev}=0)\Rightarrow \\ \displaystyle &\displaystyle \exists \circ (\exists \diamond (\mathit{Bmal}=0\wedge \mathit{Clock}\text{-}\mathit{Bmal1}=0\wedge \mathit{Per}\text{-}\mathit{Cry}=0\wedge \mathit{Rev}=0))\\ \displaystyle &\displaystyle \wedge ((\exists \diamond (\exists \square \mathit{Myc}=1\wedge \mathit{p53}=0)))\\ \displaystyle &\displaystyle \end{array}\end{eqnarray*}

*ψ*_2_ states the condition where despite of all the core clock proteins oscillating in a homeostatic manner, MYC starts over expression and p53 expression is suppressed.

(3) \begin{eqnarray*}{\psi }_{3}=((\exists \diamond \mathit{p53}=1)\Rightarrow (\exists \diamond \mathit{Myc}=0)\Rightarrow (\mathit{p53 = 0} \mathcal{U} \mathit{p53}=1)).\end{eqnarray*}

The third property *ψ*_3_ states that expression of p53 at its normal level may inhibit MYC’s over expression which further leads to decrease in p53 expression to 0 and afterwards the expression will again increase to 1.

(4) \begin{eqnarray*}{\psi }_{4}=((\exists \diamond (\forall \square \mathit{Myc}=1)))\end{eqnarray*}

*ψ*_4_ states the condition where MYC starts over expressing.

(5) \begin{eqnarray*}{\psi }_{5}=((\exists \diamond (\forall \square \mathit{p53 = 0})))\end{eqnarray*}

*ψ*_5_ states the condition where p53 is suppressed.

Due to the generation of a large number of models (≈20,000) by SMBioNet some of the parameter values were restricted based on the rules mentioned in the study of Bernot et al. (2004). The generated models were reduced down to 288 out of which 144 satisfied the CTL formula. Further reduction on the basis of biological observations reduced the number of models to four. These four models differed in the parameter values for *K*_*CB*_ and *K*_*Myc*_. Afterwards, out of these four models, a single parameter set was selected on the basis of biological knowledge for further analysis. The parameter sets generated and verified by SMBioNet and the one selected are mentioned in Table 1. The SMBioNet input and output code is provided in Supplementary Files 3 and 4, respectively. This parameter set along with BRN was used for the construction of a logical regulatory graph in GINsim (Supplementary File 2).

**Table 1:** Parameters, resource sets, parameter values provided to SMBioNet, parameter values generated by SMBioNet and the final selected set of parameters.

Parameters	Resources	Values		
		Allowed	Generated	Selected
*K*_*Bmal*_				
	{}	0	0	0
	{Rev}	1	1	1
*K*_*CB*_				
	{}	0	0	0
	{Bmal}	0,1	0,1	1
	{PC}	0	0	0
	{Bmal,PC}	1	1	1
*K*_*PC*_				
	{}	0	0	0
	{CB}	1	1	1
*K*_*REV*_				
	{}	0	0	0
	{CB}	1	1	1
*K*_*p*53_				
	{}	0	0	0
	{Bmal}	1	1	1
	{Myc}	1	1	1
	{Bmal,Myc}	1	1	1
*K*_*Myc*_				
	{}	0	0	0
	{p53}	0	0	0
	{CB}	0	0	0
	{Myc}	0,1	0	0
	{p53,CB}	0,1	0,1	1
	{Myc,p53}	0	0	0
	{Myc,CB}	0	0	0
	{Myc,CB,p53}	1	1	1

### Petri net modeling and analysis

**Figure 7:**
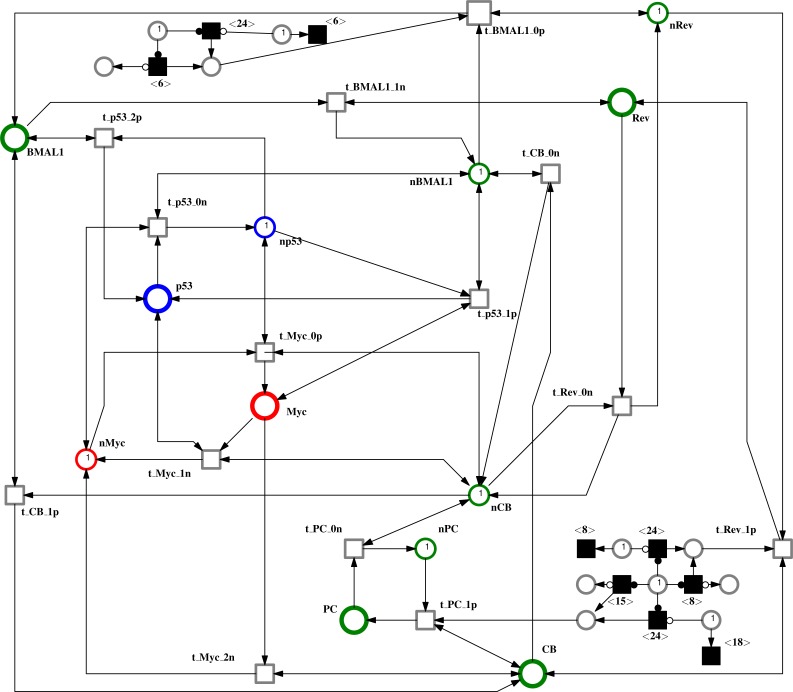
Peri net model for the system under consideration. Black filled transitions represent deterministic (delayed) transitions used to control the oscillatory timings of circadian clock proteins BMAL1, PER-CRY, CLOCK-BMAL1 and REV-ERBS. Green ‘○’ represents circadian clock protein, blue ‘○’ depicts p53 and red ‘○’ represents MYC. Each place has a complementary place labeled with ‘n’ responsible for adding and removing tokens from that place. Transition names ending with ‘p’ are responsible for activation while transition names ending with ‘n’ are responsible for inhibition of each entity.

The logical regulatory graph (BRN + logical parameters) generated in GINsim was converted to a standard discrete Petri net and finally into a Continuous Petri net (see Fig. 7) using the method described by Chaouiya, Remy & Thieffry (2006). Discrete places and transitions were converted into continuous places and transitions, respectively. Each entity had two complementary places along with two transitions labeled as p and n, regulating its activation and inhibition, respectively. Furthermore, this Petri net was modified to a Timed Continuous Petri net (TCPN). A subnet consisting of deterministic (delayed) transitions (using time delays) was added to this model and the rates for the continuous transitions were also adjusted based on delay constraints (i.e., the kinetic rate parameters were adjusted keeping in view the already reported wet lab observations about the evolution of the entities involved in the network under study). Three rate parameter sets were manually defined for the continuous transitions for each entity (Table 2). Our baseline scenario is the Normal scenario in which the rates were adjusted keeping in view their evolution sequence in the pathway. The simulation results obtained from this case are in agreement with already known experimental observations. As the expression pattern of circadian proteins: BMAL1, PER, CRY and REV-ERBs are greatly affected by jet lag (Filipski et al., 2005), therefore, to introduce the effect of mild and chronic jet lag to the baseline model, the activation and inhibition rates of the corresponding entities were altered (shown in Table 2). To lower the expression of any entity the rates for ‘p’ transitions were lowered and those for ‘n’ transitions were increased to create an inhibitory effect on the respective entity. Rates were changed qualitatively in accordance with the increasing intensity of jet lag from mild to chronic. Since we are using rates based on mass action kinetics therefore the curves are not piece-wise linear but differentiable. The Petri net model is provided as Supplementary File 1.

**Table 2:** Kinetic parameters of the mass action rates used for continuous transitions. For each entity two types of transitions (p and n) are used for activation and inhibition, respectively.

Entities	Normal		Mild		Chronic	
	Activation	Inhibition	Activation	Inhibition	Activation	Inhibition
BMAL1	1	1	0.50	1.3	0.15	2
CLOCK-BMAL1	1	1	1	1	1	1
PER-CRY	0.97	1	0.40	1.3	0.20	2
REV	0.97	1	0.40	1	0.20	1
Myc	1	1	1	1	1	1
p53	1	1	1	1	1	1

Case 1: Normal

**Figure 8:**
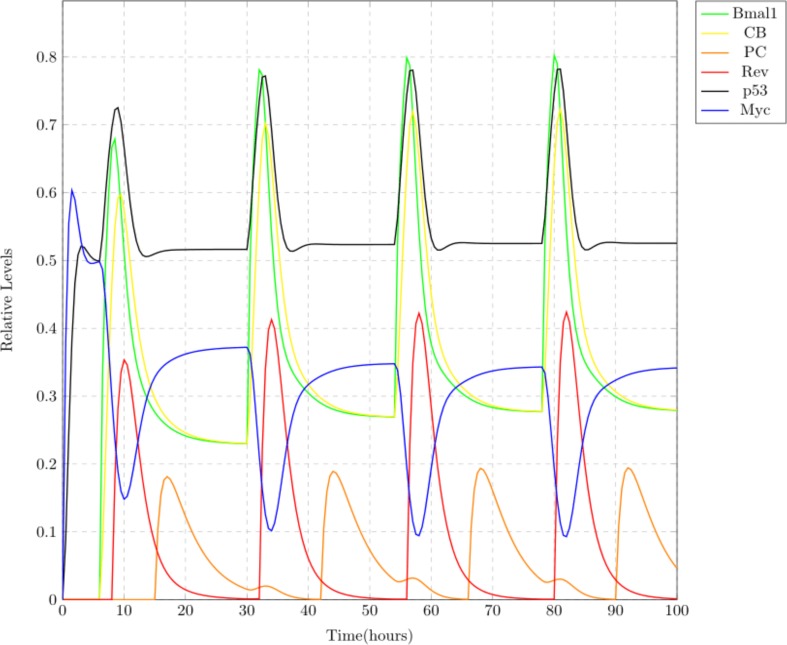
Simulation results depicting the normal oscillatory behavior of circadian clock proteins where each clock protein oscillating in a 24 h periodic manner. The core clock proteins are directly influencing the expression of MYC and p53. p53 and MYC are showing opposite behavior, i.e., with the increase in one of them, the other decreases and vice versa.

This scenario depicts the usual behavior of circadian clock. Wet lab experiments state that BMAL1 shows its maximum expression at around 0600 hrs, PER-CRY at 1,800 hrs and REV-ERBS at 0800 hrs, with a repetition after every 24 h (Relógio et al., 2011). These clock entities are showing oscillations at approximately the same timings in the simulation results shown in Fig. 8. Both the complexes CLOCK-BMAL1 and PER-CRY show oscillations in an opposite manner as the later suppresses the former. Same behavior is observed for REV-ERBs and BMAL1 proteins. MYC and p53 are co-expressed known to work in an opposite manner as p53 plays an important role in preventing MYC oncogenic expression (Lee et al., 2010; Dang, O’Donnell & Juopperi, 2005). Expression patterns of these proteins in the simulation results imitate this behavior (see Fig. 8).

Case 2: Mild

**Figure 9:**
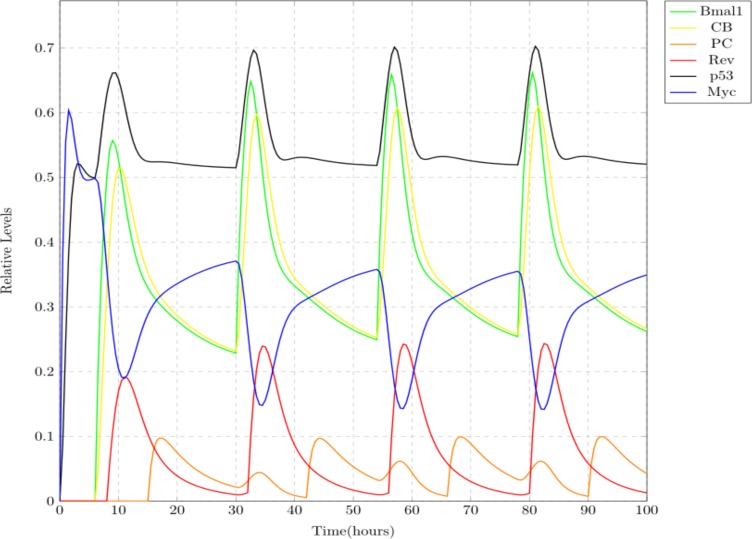
Simulation results showing mild effect of jet lag on circadian clock. Suppression in the relative expression levels of core clock proteins and p53 is clearly visible. Due to this, the inhibitory effect of p53 on MYC is reduced. These simulation results represent that there is a mild stress on the circadian system and it can be recovered.

Altered sleep/wake schedule due to traveling etc. can lead to mild jet lag effect which can cause disruptions in our circadian clock. In the model, rates of clock proteins were altered to generate a mild jet lag effect. These rates are shown in ‘Mild’ column of Table 2. Altered expression of circadian clock proteins consequently disturbed the MYC and p53 expression as shown in the simulation results (see Fig. 9). The expression of p53 was suppressed along with reduced inhibition of MYC. This disruption is mild and re-adjustment of the circadian clock through proper meal timings and sleep/wake routine (Filipski et al., 2004) might normalize MYC and p53 levels.

Case 3: Chronic

**Figure 10:**
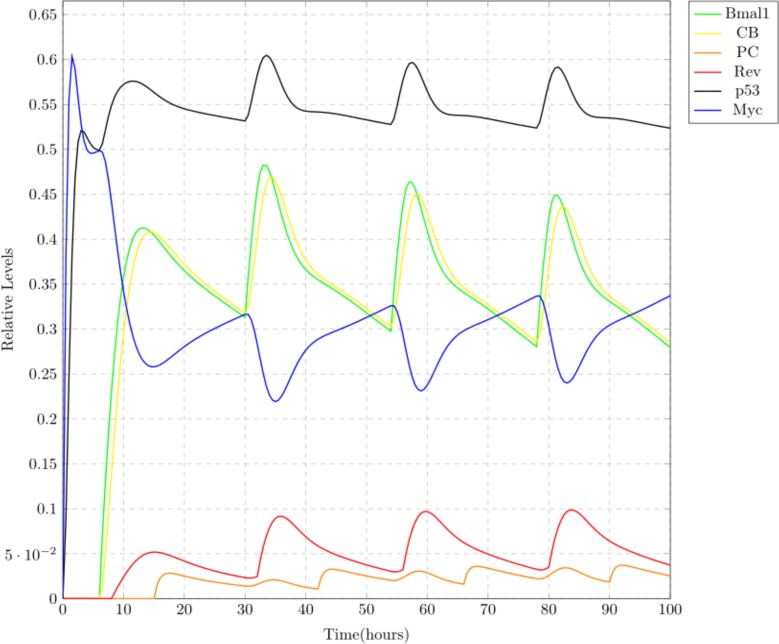
This case depicts chronic jet lag effect, where the relative expression levels of the core clock proteins are highly disturbed. This disruption lowers p53 levels causing lower inhibition of MYC. The persistently high expression of MYC can lead to growth of tumor.

Chronic disruptions in the circadian clock can be due to long term jet lag and night shift work. Jet lag produces negative impacts on each clock protein by dampening its expression levels leading to disturbed expression pattern of MYC and p53 (Filipski et al., 2004). Rates of the clock proteins in the Petri net model were altered to produce the effect of severe jet lag, as mentioned in column “Chronic” of Table 2. Simulation results shown in Fig. 10 depict that under the effect of chronic jet lag, the circadian proteins suffer from a great disruption in their expression levels. Due to this disturbance, p53 shows a suppressed pattern of expression and MYC starts expressing itself persistently (i.e., its inhibition is significantly decreased) (see Fig. 10).

### Comparison of simulations

**Figure 11:**
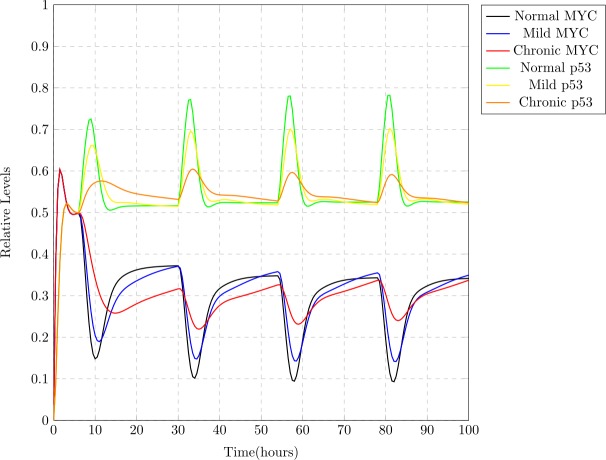
A comparison between normal, mild and chronic cases with respect to MYC and p53 levels. Notable suppression in the relative expression levels of p53 and persistent high expression of MYC protein due to jet lag can be observed.

Comparison of the the simulation results shown in Figs. 8–10 is provided in Fig. 11. It gives a clear picture of the effect of circadian disruptions on p53 and MYC. It can clearly be seen in Fig. 11 that MYC starts over expressing itself with lower inhibition whereas; clock disruption causes suppression in p53 levels. Over expression of MYC does not mean its uncontrolled expression but its persistent high expression which means that the expression level of MYC shows more fluctuations during a normal cell cycle in contrast to the tumor cells where its expression remains rather persistently high (compare the expression value in normal and chronic MYC during time frames 10–20, 30–40, 50–60 and 80–90). It can also be observed that the highest expression level attained by chronic p53 is lower as compared to the normal one (see the peaks considered as its highest expression during time frames 10–20, 30–40, 50–60 and 80–90) due to which the inhibitory effect of p53 on MYC is reduced. Thus, in case of chronic jet lag, overall the expression of MYC remains persistently at a higher level and does not undergo much fluctuations while that of p53 remains lower and cannot reach the expression levels that it attains normally. From this we can also assume that a more severe case can lead to diminished inhibition of MYC at any point. This suggests that clock disruption can lead to lower expression of tumor suppressor protein p53, causing DNA damages and persistently high expression of MYC causing abnormal cellular proliferation.

## Discussion

This section gives summary of the results mentioned in ‘Results’.

### Disruption of circadian rhythms through Jet lag

Short-term interruptions of circadian rhythms due to “jet lag” and “shift work” are known to cause metabolic and physiological disturbances. But these disruptions are reversible and clock can be readjusted to its normal timings (Erren et al., 2010). Recently, by the International Agency for Research on Cancer (IARC), long term shift work and chronic jet lag effect has been classified as a probable human carcinogen. This classification places jet lag and shift work in the same risk class as ultraviolet radiation. Exposure to artificial light conditions through shift work or jet lag disrupts the body’s ability to entrain effectively to a 24 hr time-frame which cause a phenomenon known as “light at night”. Exposure to light and darkness at unusual times leads to disruption of the normal sleep-wake rhythms. This causes desynchronization between central and the peripheral clocks. Subsequently, circadian clock outputs, which have dominant downstream effects, become disrupted. The circadian clock regulates cellular functions including cell division cycle. The circadian clock and cell cycle interact at the level of genes, proteins and biochemical signals. The cell division cycle is synchronized with the circadian clock which also helps in maintaining the integrity of the genome (Savvidis & Koutsilieris, 2012; Sahar & Sassone-Corsi, 2009).

In several studies (Fu et al., 2002; Filipski et al., 2004; Filipski et al., 2005; Yang et al., 2009; Lee et al., 2001) artificial jet lag was imposed on mice and its effect on circadian genes was observed. Jet lag caused suppressed and irregular circadian clock gene expression. As some genes between circadian clock and cell division are coupled, the alteration in circadian clock proteins directly affected the proteins involved in cell division cycle. Disruption in the expressions of circadian clock proteins lead to the abnormal division of a cell. Two main proteins found deregulated in tumors are MYC (proto-oncogene protein) and p53 (tumor suppressor). These proteins play an important part in cellular proliferation and DNA damage control. These studies show over expression of MYC and p53 suppression due to circadian clock disruption. This alteration leads to the proliferation of damaged cells as MYC is an oncogene and facilitates the growth of tumor. In addition, circadian disruption compromises the behavior of p53 thus affecting its DNA repair process. (Fu et al., 2002; Filipski et al., 2004; Filipski et al., 2005).

In this study, the connection of circadian clock with MYC and p53 was modeled using Petri net framework (Fig. 7). Simulation results shown in Figs. 8–10 depict three different case studies of jet lag disrupted circadian clock. These results are in accordance with the above mentioned observations. The first case (see Fig. 8) shows the normal behavior of an undisrupted clock with the usual oscillatory behavior of each protein. These results show that an undisrupted clock will oscillate in its usual manner and consequently the coupled proteins MYC and p53 also oscillate in their specific periodic manner. The second case (Fig. 9) describes a situation where circadian clock proteins are experiencing a slight suppression which is due to a mild jet lag effect. Mild suppression of clock proteins slightly affected MYC and p53 expression pattern. The last case (Fig. 10) describes the chronic effect of jet lag, i.e., jet lag for a long period of time as occurs in the case of frequent travelers or night shift workers. Resulting simulations clearly show over expression of MYC and suppression of p53 due to disruptions in clock proteins. Disturbances in the expression pattern of these vital cell cycle proteins can effect the normal cell cycle. Suppression of p53 leads to the failure of its DNA repair activity causing abnormality in the cells and persistent expression of MYC supports the proliferation of abnormal cells (Filipski et al., 2004; Filipski et al., 2005).

## Conclusion

Circadian genes are involved in the regulation of different vital metabolic processes such as cell division. Longterm jet leg effect can disrupt the circadian clock organization, thus, causing deregulated cellular proliferation and tumor growth. This study employed various formal methods which start with model checking for the inference of logical parameters. These parameters are then used for the generation of a logical regulatory graph which is finally converted to to the Petri net model to obtain deep insights into the circadian system and analyze its behavior in normal and jet lag conditions. The model presented in this study depicts that circadian clock plays a dual role in cell cycle progression. On one hand, it controls the expression of oncogenic protein MYC while on the other hand it suppresses proliferation of damaged cells by regulating the activation of p53. Keeping in view the simulation results obtained after modeling the effects of jet lag on circadian clock, it can be stated that alterations in sleep/wake and light/dark cycles cause circadian disruption. This disruption negatively impacts the expression of vital cell cycle genes MYC and p53. The expression levels of p53 are suppressed resulting in persistently high expression of MYC. This condition favors the proliferation of tumor cells. Therefore, it is concluded that a properly functioning circadian clock is necessary for ensuring a tumor free system.

##  Supplemental Information

10.7717/peerj.4877/supp-1 Supplemental Information 1 Supplementary files Initially, parameter sets are generated through SMBioNet by providing it all the variables and the regulatory interactions of the BRN. From the generated parameter sets, a single set is selected. The selected parameter set was used to generate a qualitative model in GINsim. This model was then converted into a Petri net model which was primarily a discrete model and was further converted into Hybrid PN.
**Supplementary File 1- Petri Net Model:** A Hybrid PN with 3 parameter sets to model the normal scenario, mild jetlag and chronic jetlag. (Use Snoopy to open this file.)
**Supplementary File 2-GINsim Model:** The Qualitative Model generated using the BRN and the verified parameter values. (Use GINsim to open this file.)
**Supplementary File 3-SMBioNet Input Code:** This code consists of: Variables, Parameters, Regulatory interactions and CTL formulas This was provided to SMBioNet for the generation of parameter sets (Use notepad to view).
**Supplementary File 4- SMBioNet Output Code:** The output file generated by SMBioNet consisting of all the generated models (Use MS Word to view).
**Supplementary File 5- Additional Information**. File having some explanation regarding how were the kinetic rate parameters defined? And some additional information about Fig. 7. (Use MS Word to view).****
**Supplementary File 6- THPN example file.** Petri net file for THPN example shown in Fig. 6. (Use Snoopy to open this file.)
**Supplementary File 7- Simulation for the THPN example.** Simulation diagram of the THPN example shown in Fig. 6 with a brief explanation. (Use MS Word to view).

## Additional Information and Declarations

### Data Availability

The following information was supplied regarding data availability: The raw data are included in the Supplemental Files.
