# Supplementary material for: Modeling and analysis of the impacts of jet lag on circadian rhythm and its role in tumor growth
Source: PeerJ. 2018 Jun 6;6:e4877. doi: 10.7717/peerj.4877 (PMC5994163; doi:10.7717/peerj.4877)
Supplement: Supplemental Information 1 — Initially, parameter sets are generated through SMBioNet by providing it all the variables and the regulatory interactions of the BRN. From the generated parameter sets, a single set is selected. The selected parameter set was used to generate a qualitative model in GINsim. This model was then converted into a Petri net model which was primarily a discrete model and was further converted into Hybrid PN. Supplementary File 1- Petri Net Model: A Hybrid PN with 3 parameter sets to model the normal scenario, mild jetlag and chronic jetlag. (Use Snoopy to open this file.) Supplementary File 2-GINsim Model: The Qualitative Model generated using the BRN and the verified parameter values. (Use GINsim to open this file.) Supplementary File 3-SMBioNet Input Code: This code consists of: Variables, Parameters, Regulatory interactions and CTL formulas This was provided to SMBioNet for the generation of parameter sets (Use notepad to view). Supplementary File 4- SMBioNet Output Code: The output file generated by SMBioNet consisting of all the generated models (Use MS Word to view). Supplementary File 5- Additional Information. File having some explanation regarding how were the kinetic rate parameters defined? And some additional information about Fig. 7. (Use MS Word to view). Supplementary File 6- THPN example file. Petri net file for THPN example shown in Fig. 6. (Use Snoopy to open this file.) Supplementary File 7- Simulation for the THPN example. Simulation diagram of the THPN example shown in Fig. 6 with a brief explanation. (Use MS Word to view). [file peerj-06-4877-s001.zip › Supplementary Files/Supplementary5.docx]

How were the kinetic rate parameters defined?

The rates shown in Table 2, are kinetic rate parameters for mass action kinetics and were adjusted manually as follows:

- Rates of the first parameter set, mentioned in the column “Normal” were adjusted keeping in the view the delay constraints based on biological knowledge, in order to produce simulations that mimic the behavior already mentioned in the reported literature (i.e. timings and the pattern followed by entities). As this parameter set produced results that were mimicking the already reported observations, it was used as a baseline scenario.
- To introduce the effect of mild and chronic jetlag to the baseline model, the activation and inhibition rates of the jetlag influenced entities were altered (shown in Table 2). The activation rates were lowered and inhibition rates were increased to lower the expression of these entities, as happens in the case of jetlag. Rates were changed in accordance with the increasing intensity of jetlag from mild to chronic.

Some additional information about Figure 7.

Places and transitions of different size and color were used only to make it more understandable visually. Only those places aren’t labeled which are used in subnets (as they are not representing any specific entity).

The arcs with a standard arrow at one end and the hollow on the other end are actually two arcs, the standard one for activation and the hollow one showing inhibition. These arcs represent that one entity (at the starting point of the standard arc) is activating the entity at the end point of the standard arc and this entity after being activated inhibits the entity that activated it in the first place (present at the end of inhibitory arc).

The subnets are used to produce periodic timely oscillations of the circadian clock entities. These subnets were introduced manually into Petri net after conversion from the qualitative model and have not been obtained from GINSim. Subnets work in a way so that all the circadian clock entities show peaks after every 24 hours and on the time that is already reported in the published literature.
