# Supplementary material for: Modeling and analysis of the impacts of jet lag on circadian rhythm and its role in tumor growth
Source: PeerJ. 2018 Jun 6;6:e4877. doi: 10.7717/peerj.4877 (PMC5994163; doi:10.7717/peerj.4877)
Supplement: Supplemental Information 1 — Initially, parameter sets are generated through SMBioNet by providing it all the variables and the regulatory interactions of the BRN. From the generated parameter sets, a single set is selected. The selected parameter set was used to generate a qualitative model in GINsim. This model was then converted into a Petri net model which was primarily a discrete model and was further converted into Hybrid PN. Supplementary File 1- Petri Net Model: A Hybrid PN with 3 parameter sets to model the normal scenario, mild jetlag and chronic jetlag. (Use Snoopy to open this file.) Supplementary File 2-GINsim Model: The Qualitative Model generated using the BRN and the verified parameter values. (Use GINsim to open this file.) Supplementary File 3-SMBioNet Input Code: This code consists of: Variables, Parameters, Regulatory interactions and CTL formulas This was provided to SMBioNet for the generation of parameter sets (Use notepad to view). Supplementary File 4- SMBioNet Output Code: The output file generated by SMBioNet consisting of all the generated models (Use MS Word to view). Supplementary File 5- Additional Information. File having some explanation regarding how were the kinetic rate parameters defined? And some additional information about Fig. 7. (Use MS Word to view). Supplementary File 6- THPN example file. Petri net file for THPN example shown in Fig. 6. (Use Snoopy to open this file.) Supplementary File 7- Simulation for the THPN example. Simulation diagram of the THPN example shown in Fig. 6 with a brief explanation. (Use MS Word to view). [file peerj-06-4877-s001.zip › Supplementary Files/Supplementary7.docx]

Simulations of the Example THPN shown in Figure 6.


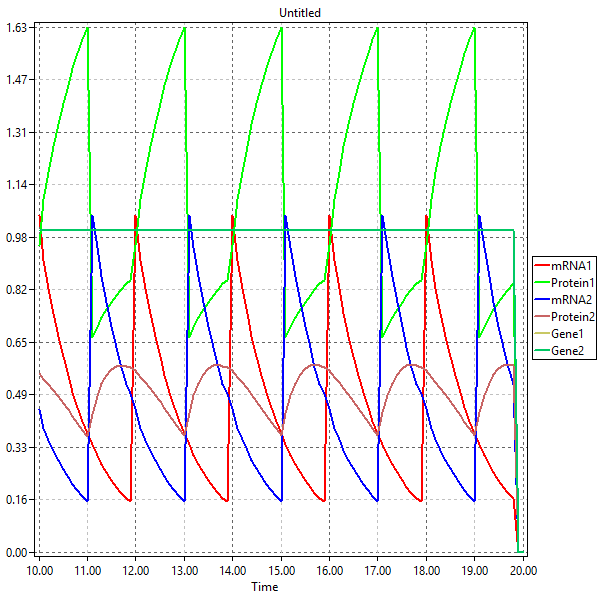


In these simulation results, Gene1 and Gene2 are constant at discrete level 1, due to read arcs connecting these places to the transitions. mRNA1 and mRNA2 showing oscillations in accordance with Protein1 and Protein2. Both mRNA1 and mRNA2 are in anti-phase to their respective proteins. Similar behavior is observed in the long run but a specific interval (10-20) is shown to make it clearer.
